# Identification of tumour-associated and germ line p53 mutations in canine mammary cancer

**DOI:** 10.1038/sj.bjc.6690709

**Published:** 1999-10

**Authors:** N Veldhoen, J Watterson, M Brash, J Milner

**Affiliations:** YCR P53 Research Group, Department of Biology, University of York, York, YO10 5DD, UK; Battle Flatts Veterinary Clinic, 38 Main Street, Stamford Bridge, York, YO4 1AB, UK

**Keywords:** germline, DNA mutation, canine p53, mammary cancer

## Abstract

Mutations of the tumour suppressor p53 gene are found in a number of spontaneous canine cancers and may contribute to increased cytogenetic alterations and tumour formation. Using reverse transcription and DNA amplification, we isolated p53 cDNA from normal and tumour tissue of ten canine mammary cancer patients. DNA sequencing identified p53 mutations in three of the ten patients. These included tumour-associated p53 gene mutations within exons 2 and 5 and a germ line deletion of exons 3 to 7. These results support a role for p53 inactivation in canine mammary tumour formation and breed predisposition to cancer. Such information could prove invaluable in the successful outbreeding of inherited predisposition to cancer in the dog. A putative polymorphism was also identified at codon 69 in exon 4 and we discuss the possibility that similar polymorphisms may be associated with human breast cancer. © 1999 Cancer Research Campaign


*British Journal of Cancer* (1999) **81**(3), 409–415
© 1999 Cancer Research Campaign
Article no. bjoc.1999.0709

### Identification of tumour-associated and germ line p53 mutations in canine mammary cancer

**N Veldhoen[1], J Watterson[1], M Brash[2] and J Milner[1]**

[1]YCR P53 Research Group, Department of Biology, University of York, York YO10 5DD, UK; [2]Battle Flatts Veterinary Clinic, 38 Main Street, Stamford Bridge, York YO4 1AB, UK

**Summary** Mutations of the tumour suppressor p53 gene are found in a number of spontaneous canine cancers and may contribute to increased cytogenetic alterations and tumour formation. Using reverse transcription and DNA amplification, we isolated p53 cDNA from normal and tumour tissue of ten canine mammary cancer patients. DNA sequencing identified p53 mutations in three of the ten patients. These included tumour-associated p53 gene mutations within exons 2 and 5 and a germ line deletion of exons 3 to 7. These results support a role for p53 inactivation in canine mammary tumour formation and breed predisposition to cancer. Such information could prove invaluable in the successful outbreeding of inherited predisposition to cancer in the dog. A putative polymorphism was also identified at codon 69 in exon 4 and we discuss the possibility that similar polymorphisms may be associated with human breast cancer. © 1999 Cancer Research Campaign

**Keywords:** germline; DNA mutation; canine p53; mammary cancer

Point mutation or deletion/insertion within the p53 tumour suppressor gene has been estimated to occur in over half of all human cancers and may directly contribute to tumour progression (Chang et al, 1994; Wada et al, 1994; Hainaut et al, 1997). A number of studies indicate that the canine p53 gene is also mutated in tumour cells from patients presenting with spontaneous cancers of different types. These include: thyroid carcinoma (Devilee et al, 1994), oral papilloma (Mayr et al, 1994), osteosarcoma (van Leeuwen et al, 1997; Johnson et al, 1998), circumanal gland adenoma (Mayr et al, 1997), lymphoma (Veldhoen et al, 1998) and mammary tumours (van Leeuwen et al, 1996; Chu et al, 1998; Mayr et al, 1998). The apparent similarity of p53 inactivation in tumours of both humans and dogs suggests that investigations into the molecular aetiology of canine cancer may help to further our understanding of p53-related neoplasia.

Mammary cancer represents the most common malignant tumour in female dogs and certain breeds appear to display an increased cancer predisposition (MacVean et al, 1978; Priester and McKay, 1980). In order to investigate the role of p53 in mammary tumour formation and progression, we determined the p53 status in ten canine cancer patients. Two patients were found to have tumour-associated p53 mutations, while a third contained germ line modifications of the p53 gene. These results indicate that both spontaneous and inherited alterations within the p53 gene are associated with canine mammary cancer. In addition, the identification of germ line p53 gene mutations associated with canine cancer (this study and Veldhoen et al, 1998) lends support for a critical role for p53 inactivation in breed predisposition to cancer.

*Received 10 December 1998*
*Revised 23 March 1999*
*Accepted 1 April 1999*

*Correspondence to:* J Milner

### MATERIALS AND METHODS

### Plasmids and bacterial strains

The pK9 plasmid contains the wild-type canine p53 cDNA cloned into pLitmus 29. Canine p53 cDNA fragments isolated from surgically removed normal and tumour tissue samples of each patient were cloned into pBluescript SK+. Positively identified mutant p53 cDNA fragments from patients 5 and 6 were subcloned into the *Sac*I and *Mlu*I sites of pK9 to produce full-length p53 mutant constructs pMP5 and pMP6 respectively. All plasmids were maintained in *Escherichia coli* XL-1 Blue MRF'.

### RNA isolation and p53 cDNA amplification

Total RNA was prepared from a 100 mg sample of normal and/or tumour tissue from each patient using the RNeasy RNA isolation kit as per the manufacturer's protocol (Qiagen). Note that normal tissue was not available for patient 6. Complementary DNA fragments containing p53 coding sequence were amplified from 2 μg total RNA using the Access RT-PCR system as per the manufacturer's protocol (Promega). The primers used in the reaction were NTup (5′GCGGTACCCAATGGAGGAGTCGCAGTCAGAG3′) and CTdn (5′GCGGATCCTTAACCTACGTCTGAGTCAAGCC-CTTCTCTC3′) which amplify the complete open reading frame of canine p53. The thermocycle programme included a first strand cDNA polymerization step of 48°C for 45 min and 94°C for 2 min, then 40 cycles of 94°C (30 s), 50°C (1 min), 68°C (2 min) and a final polymerization step of 68°C for 7 min.

### Secondary DNA amplification and p53 cDNA cloning

A second nested DNA amplification reaction was performed to increase the yield of p53 cDNA product. This reaction used the primers *Sac*Iup (5′CAGTCAGAGCTCAATATCGACCCCCCTCT-

**Table 1** Clinical information on the canine mammary cancer patients

| Patient number | Age (years) | Breed | Intact (I)/ Neutered (N) | Histopathology | Prognosis |
|---|---|---|---|---|---|
| 1 | 10 | Collie cross | I | Carcinoma | Guarded |
| 2 | 12 | Spaniel | I | Carcinoma | Guarded |
| 3 | 8 | Lurcher | I | Carcinoma | Guarded |
| 4 | 10.5 | Border collie | I | Carcinoma | Guarded |
| 5[a] | 10 | Labrador | I | Carcinoma | Guarded |
| | | | | Adenoma | Guarded |
| 6[b] | – | – | – | – | – |
| 7 | 9 | Collie cross | N | Adenoma | Good |
| 8 | 11 | Old English sheepdog | I | Adenoma | Good |
| 9 | 5 | Boxer | N | Adenoma | Good |
| 10 | 17 | Jack Russell terrier | I | Sarcoma | Guarded |

[a]Note: Patient number 5 presented with two discrete mammary tumours. [b]No clinical information was available for this patient.

GAGCCAGGAGACATTT3′) and 9dn (5′GCGGATCCTGA-AGGGTGAAATATTC3′) and amplified a segment of the canine p53 cDNA containing exons 2 to 9. The 50-µl amplification reaction included PC2 buffer (50 mM Tris–HCl pH 9.1, 16 mM $(NH_4)_2SO_4$, 3.5 mM magnesium chloride, 150 µg $ml^{-1}$ bovine serum albumin (BSA), 12.5 pmol of each primer, 200 µM dNTPs (dATP, dCTP, dGTP, dTTP)), 1 µl of first round RT-PCR sample and 1.5 units *Taq* Supreme (Helena BioSciences). The thermocycle programme was identical to that used for the first round reverse transcription polymerase chain reaction (RT-PCR) without the initial polymerization step of 48°C for 45 min.

Canine p53 cDNA fragments isolated from each patient were digested with the restriction endonucleases *Sac*I and *Bam*HI, separated on a 1% agarose gel, and isolated by excision and spin column centrifugation (Pharmacia). Purified p53 cDNA was then ligated into pBluescript SK+. Positive plasmid clones were identified by restriction analysis and DNA amplification using conditions identical to those above and including the primers 5/6up (5′CGGAATTCCTCCCCTCTCCTCACCAAG3′) and 8dn (5′GCGGATCCTCGCTTGGTACTCCCGGGGG3′). A minimum of 14 individual p53 cDNA clones derived from each patient tissue sample were pooled for sequence analysis. The cloned p53 cDNA was sequenced using a T7 sequencing kit as per the manufacturer's protocol (Pharmacia Biotech).

### Amplification of the p53 gene deletion from patient 9 genomic DNA

Genomic DNA was prepared from normal and tumour tissue of patient 9 using a genomic DNA miniprep kit as per the manufacturer's protocol (Immunogen International). DNA fragments containing the p53 gene were amplified using the primer pairs *Sac*Iup:9dn, 5/6up:7dn (5′GCGGATCCTGGAGTCTTCCAGG-GTGAT3′), P9.1 (5′CAGAATTGTGGAACCTGTAAGT3′):9dn, and P9.2 (5′GGCTCCCCACCAGCCCTCTGGG3′):9dn in a 50-µl reaction containing PC2 buffer, 12.5 pmol of each primer, 50 µM dNTPs, 320 ng genomic DNA, 5 units *Taq* Supreme (Helena BioSciences). The thermocycle programme included a denaturation step of 94°C (7 min), 40 cycles of 94°C (1 min), 62°C (30 s), 68°C (1.5 min), and a final polymerization step of 68°C for 7 min. The amplified DNA products were electrophoresed on a 1% agarose gel. DNA fragments amplified using the *Sac*Iup and 9dn primers were cut with *Sac*I and *Bam*HI and cloned into the identical sites of pBluescript SK+. Positive clones were sequenced using a T7 sequencing kit as per the manufacturer's protocol (Pharmacia Biotech).

### Transcription and translation

Plasmids pMP5 and pMP6 containing mutant p53 cDNA isolated from patients 5 and 6, respectively, were digested with *Eco*RI. Plasmid pK9 containing wild-type canine p53 cDNA was similarly digested with *Eco*RI. RNA transcription was performed as described by Veldhoen and Milner (1998). Wild-type and mutant canine p53 proteins were translated at 30°C in a rabbit reticulocyte lysate system as described by Gamble and Milner (1988). The efficiency of translation was determined by TCA precipitation on glass filters followed by scintillation counting.

### Immunoprecipitation

Protein conformation was assessed by immunoprecipitation with the anti-p53 antibodies PAb240, and PAb421 as described by Cook and Milner (1990). Antibody PAb416, directed towards the large T-antigen of SV40, was used as a negative control. Immunoprecipitated proteins were resolved by 15% sodium dodecyl sulphate polyacrylamide gel electrophoresis (SDS-PAGE) and visualized by autoradiography with Fuji RX film at room temperature.

### RESULTS

### Identification of tumour-associated p53 gene mutations

Table 1 shows the clinical information for each patient analysed in this study. The status of the p53 gene in the ten patients was assessed following routine surgical removal of mammary tumour material and surrounding normal tissue. Appropriate precautions were taken to prevent cross-contamination between the two tissue types during the surgical procedure. (Note: normal tissue was not available for patient 6.) Total RNA was isolated from both normal and tumour tissue and p53 cDNA prepared by nested RT-PCR. The p53 cDNA was subsequently cloned and sequenced. Three of the dogs (patients 5, 6, and 9) were found to have p53 mutations while the remaining seven patients exhibited wild-type p53 cDNA sequence. The p53 mutations observed for patients 5, 6, and 9 were confirmed by a second independent round of cDNA isolation

 © 1999 Cancer Research Campaign

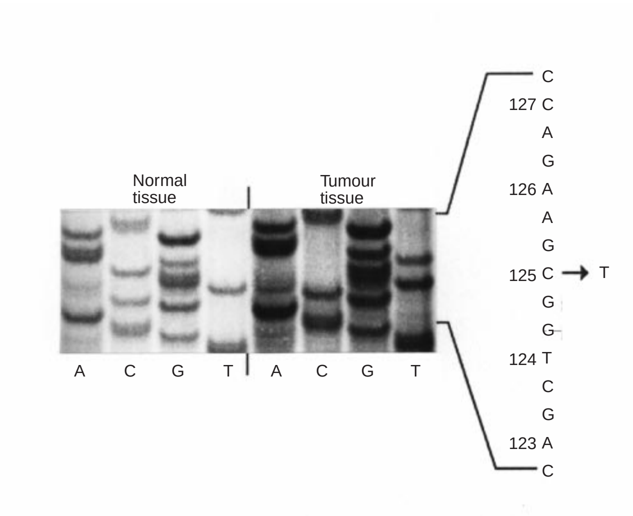

**Figure 1** Identification of a mutant p53 allele within the solid mammary carcinoma from patient 5. The exon 5 p53 cDNA sequence from normal and tumour tissue is shown along with the non-coding strand sequence substitution. Codon positions are shown to the right of the DNA sequence

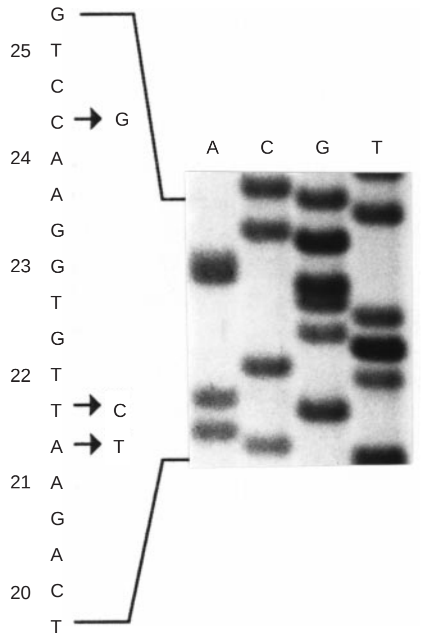

**Figure 2** Identification of a tumour-associated mutant p53 allele within patient 6. The exon 2 p53 cDNA sequence from mammary tumour tissue is depicted along with the non-coding strand sequence substitutions. Codon positions are shown to the left of the DNA sequence and sequencing. All codon positions refer to the canine p53 open reading frame sequence (Genbank Accession AF060514). Where necessary, the corresponding human p53 codon positions are also identified.

Patient 5 presented with two discrete mammary tumours; a solid carcinoma and an adenoma (Table 1). The adenoma tissue contained wild-type p53, while a p53 gene mutation was identified in the solid carcinoma tumour. This point mutation at codon 125 (GCG to GTG) within exon 5 results in a substitution from alanine to valine (Figure 1) and corresponds to the temperature-sensitive mutants Ala135Val of murine p53 (Martinez et al, 1991) and Ala138Val of human p53 (Hirano et al, 1995; Yamato et al, 1995). Mutation at the equivalent position in the human p53 gene has been observed in three breast cancer patients (Moll et al, 1992; Faille et al, 1994). Although not directly investigated, the lack of detectable wild-type p53 sequence within the carcinoma of patient 5 indicates that allelic loss of the p53 gene may have also occurred during tumorigenesis (Figure 1).

Patient 6 displayed multiple substitution mutations clustered within exon 2 of the p53 gene (Figure 2). These point mutations included substitutions of glutamic acid to aspartic acid at codon 21 (GAA to GAT), a silent mutation of the leucine residue at codon 22 (TTG to CTG), and a substitution of asparagine to lysine at codon 24 (AAC to AAG). A fourth base pair change located within exon 4 of the same p53 allele was also observed at codon 69 (CCC to CTC) resulting in a substitution of proline for leucine (not shown). Although relatively rare, multiple p53 gene mutations have been identified in two human breast cancer patients (Glebov et al, 1994; Seth et al, 1994). No wild-type p53 cDNA sequence was isolated from the tumour of patient 6 suggesting a loss of the second p53 allele (Figure 2).

The analysis of patient 9 proved to be very interesting and revealed mutations within the p53 gene isolated from both normal tissue and the mammary adenoma tumour. This strongly supports a germ line origin for these p53 gene alterations. One p53 allele was found to contain a large deletion of DNA sequence that included exons three through seven (Figure 3A). The presence of the internal p53 deletion was confirmed by sequencing both cDNA and genomic DNA clones derived from normal and tumour tissue. Mutant-specific amplification of genomic DNA isolated from normal and tumour tissue of patient 9 also points to a germ line origin for the p53 deletion mutation (Figure 3B, compare lanes 5 and 6 with lanes 8 and 9). The chromosome breakpoint within this p53 ‘mini gene’ is shown in Figure 3A and results in a fusion of introns 2 and 7 of the canine p53 gene. Analysis of the canine p53 gene sequence (Chu et al, 1998) suggests that this deletion may have originated during DNA replication through a slipped-strand mispairing mechanism (for example see Huang et al, 1994). This form of DNA damage would involve two imperfect direct repeat sequences located within introns 2 (GGACCCCTGC) and 7 (GGCCCCCTGC) of the canine p53 gene and result in loss of the 3′ repeat sequence and intervening DNA (see Figure 3A).

The second intact p53 allele of patient 9 contained a single base pair substitution at codon 69 (CCC to CTC) resulting in replacement of proline with leucine (Figure 4). This Pro69Leu substitution was observed in both normal and tumour tissue and could potentially represent a second germ line p53 mutation identified in the same cancer patient. A similar germ line mutation of the corresponding position in human p53 (Pro82Leu) has been suggested to influence the development of human breast cancer (Sun et al, 1996). An alternative explanation is that Pro69Leu may represent a polymorphism in the canine p53 gene. This is suggested by the presence of this substitution in the tumour of patient 6. Unfortunately, no normal tissue from patient 6 was available for analysis and the nature of this proline to leucine substitution at codon 69 remains unclear. In human cancers, the contribution of different human p53 polymorphic variants to tumour development remains poorly defined. However, evidence does exist for an association between the polymorphic status at Pro72Arg and human cervical cancer (Storey et al, 1998).

© 1999 Cancer Research Campaign 

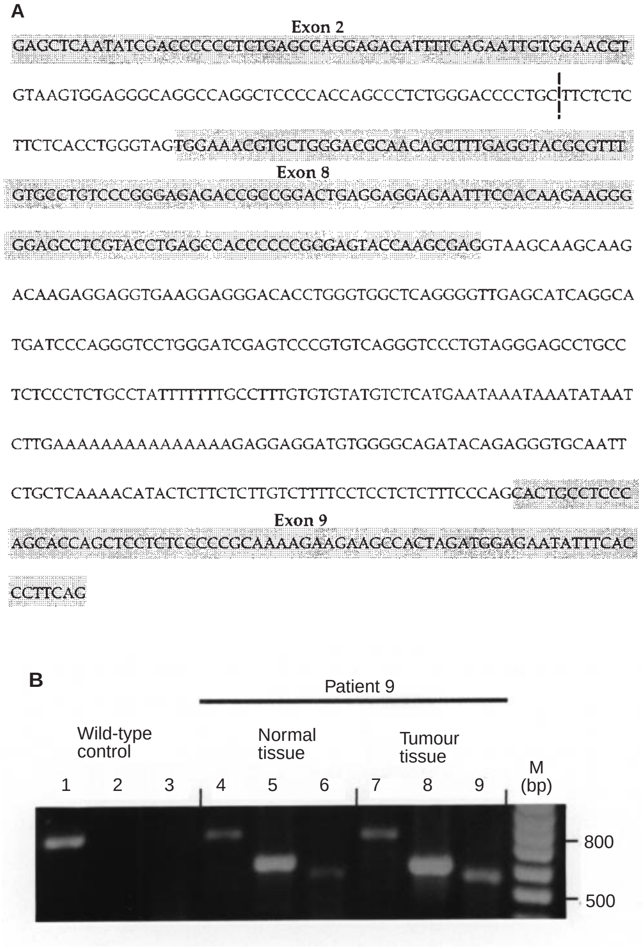

**Figure 3** Characterization of a germline deletion within one p53 allele of patient 9. (**A**) DNA sequence of the p53 allele from patient 9 containing a deletion of exons three to seven. Exon sequences are shaded. The dashed vertical line shows the proposed location of the DNA deletion event. (**B**) Direct detection of the p53 deletion mutation within genomic DNA isolated from normal (lanes 5 and 6) and tumour (lanes 8 and 9) tissue. Amplification of exons 5–7 from the second intact p53 allele isolated from normal and tumour tissue is also shown (lanes 4 and 7). Genomic DNA isolated from a healthy dog was included as a wild-type control (lanes 1–3)

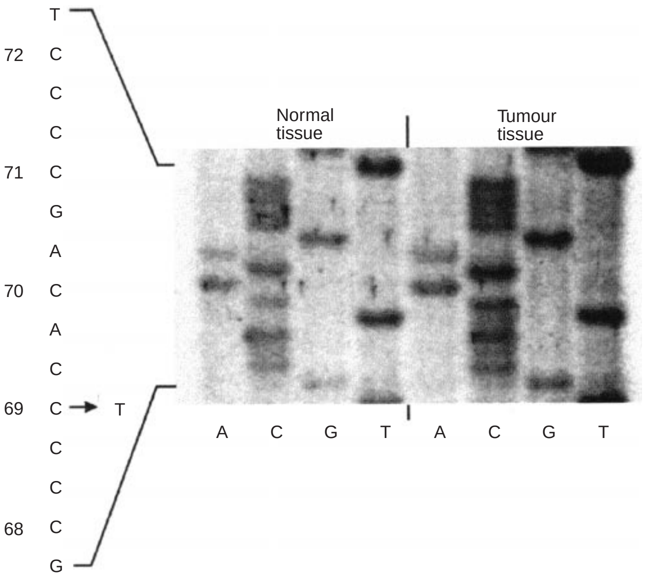

**Figure 4** Location of a single base pair substitution within the intact p53 allele of patient 9. DNA sequence from exon 4 of the p53 gene isolated from normal and tumour tissue is shown. The C to T transition at codon 69 is depicted. Codon positions are shown to the left of the non-coding strand DNA sequence

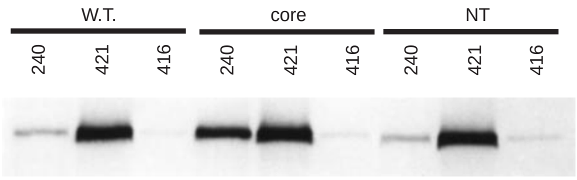

**Figure 5** Conformation of mutant canine p53 protein. Radiolabelled wild-type and mutant p53 proteins were expressed in vitro and immunoprecipitated using antibodies 240 and 421. Antibody 416 was included as a negative control. Antibody-bound p53 protein was resolved on a 15% SDS-PAGE. Mutant p53 derived from patient 5 is labelled 'core', while patient 6 mutant p53 is identified by 'NT'. Minor differences in the migration of the NT mutant protein compared with wild-type p53 may be due to the location of amino acid substitutions within the transactivation domain of p53

### Conformation of mutant p53 proteins

A large number of tumour-associated p53 mutations found in human cancer affect the conformation of the p53 protein resulting in reactivity with antibody 240 (Gannon et al, 1990). These PAb240-positive mutations are clustered within the flexible central core domain of the p53 protein. In order to determine if the location of point mutations within canine p53 affects protein conformation, we performed in vitro translation and immunoprecipitation analyses using the p53 mutants isolated from patients 5 and 6. The Ala125Val mutation of patient 5 is located within the core domain of p53, whereas the multiple p53 mutations identified in patient 6 reside within the N-terminal region. Each mutant p53 cDNA produced full length protein when expressed in vitro (Figure 5). Significant reactivity with antibody 240 was observed for the core mutant (Ala125Val) when compared with wild-type canine p53 protein (Figure 5). In contrast, no change in antibody 240 reactivity was seen for the N-terminal mutant p53 protein (Figure 5). These results demonstrate, for the first time, that the position of codon substitution in canine p53 affects protein conformation in a manner similar to that observed for human p53.

### DISCUSSION

Mutations within the p53 gene are observed in approximately 25% of human breast cancer patients and may be associated with poor prognosis and shorter overall survival rates (Casey et al, 1996; Béroud and Soussi, 1998). Recently, similar p53 mutations have been identified in primary mammary tumours of canine patients. These include point mutations at codons 236, 245 and 249 (human codon positions) within exon seven of the p53 gene (van Leeuwen et al, 1996; Mayr et al, 1998). Non-sense, frame-shift, and splice-site p53 mutations have also been described in canine mammary cancer (Chu et al, 1998). In the present study, we have identified three out of ten patients that exhibit alterations within the p53 gene isolated from primary mammary tumour tissue. These p53 mutations were located within exons two, four and five (see Figure 6).

 © 1999 Cancer Research Campaign

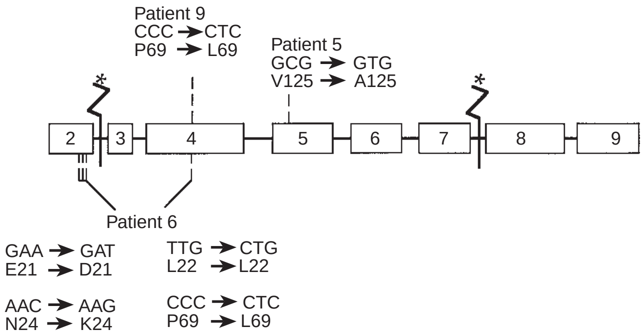

**Figure 6** Summary of DNA sequence changes identified in the p53 gene of three canine mammary cancer patients. The position of each point mutation is depicted by dashed lines. The location of DNA break points within one p53 allele of patient 9 is shown by an asterisk

Although the number of canine patients analysed was small, the frequency of p53 mutation (30%) appears similar to that observed in human breast cancer patients.

Patient 5 presented with two discrete mammary tumours that included an adenoma and a solid carcinoma which displayed a high metastatic potential. The Ala125Val mutant p53 gene was isolated from the carcinoma indicating that the more aggressive mammary tumour of patient 5 was associated with a point mutation of the canine p53 gene. Such an association between p53 mutation and high grade tumours has been noted previously in human breast cancer patients (Faille et al, 1994; Berns et al, 1996; Hartmann et al, 1997). The observed C to T transition mutation of patient 5 may have been the result of de-amination of 5-methylcytosine at the CpG position within codon 125 (GCG). In the human p53 gene, CpG dinucleotides within exons 5–8 are completely methylated in a tissue-independent manner and are, therefore, vulnerable to this form of DNA damage (Tornaletti and Pfeifer, 1995). A similar situation is likely to exist for the canine p53 gene.

The Ala125Val p53 protein translated in vitro displayed an increased mutant conformation as determined by antibody 240 reactivity. This is consistent with a number of human p53 mutants that contain amino acid substitutions within the highly conserved central core domain (for example, see Rolley et al, 1995). Adoption of the mutant conformation is suggested to contribute to nuclear accumulation of mutant p53 protein within tumour cells (Faille et al, 1994). Indeed, the corresponding human p53 mutant, Ala138Val, has been shown to display an increase in nuclear p53 protein levels within breast cancer cells (Moll et al, 1992; Faille et al, 1994). Thus, it is likely that Ala125Val p53 would show an increase in protein stability within the mammary carcinoma of patient 5.

In some instances, the human p53 gene can be the target of multiple mutations that arise from 'stuttering' of the DNA polymerase during replication (for examples see Strauss, 1997). In 20% of these cases, at least one of the base pair substitutions is silent (Strauss, 1997). In the present study, the p53 allele from patient 6 contained a cluster of three point mutations at codons 21, 22 and 24 that included a silent codon substitution at Leu22 (TTG to CTG) (see Figure 6). The identification of this mutant p53 sequence indicates that a similar 'hypermutation' mechanism exists in dogs and may target the canine p53 gene.

Two human breast cancer patients have been identified with multiple p53 mutations clustered within the central core domain of p53 (Glebov et al, 1994; Seth et al, 1994). In contrast, the mutations within canine p53 derived from patient 6 are clustered within the N-terminal domain of the protein. This region of p53 (including residues Phe19, Leu22, Trp23, and Leu26) is crucial for both transactivation activity and MDM2 binding and is highly conserved between human and canine p53 proteins (Lin et al, 1995; Kussie et al, 1996: Veldhoen and Milner, 1998). It is interesting to note that the cluster of p53 mutations identified in the tumour of patient 6 (see Figure 6) overlaps this critical region for p53 protein function. Two of these mutations (Glu21Asp and Leu22Leu) are conservative in nature and it remains unclear as to whether they would significantly alter p53 protein activity in vivo.

Inherited p53 gene mutations have been linked to the Li–Fraumeni syndrome and familial cancer predisposition (for a review see Malkin, 1994; Birch et al, 1998). Studies performed in mouse models also indicate that loss or inactivation of only one p53 allele is sufficient to promote cancer formation (Venkatachalam et al, 1998). Due to the apparent breed predisposition to cancer observed in dogs (see Priester and McKay, 1980), we have determined the status of the p53 gene in both normal and tumour cells from canine patients. The identification of p53 mutations in normal tissue strongly supports a germ line origin for these alterations in the p53 gene. At present, two patients have been identified as germline carriers for p53 mutations. These include a Bull Mastiff with lymphoma containing a frame-shift p53 mutation (Veldhoen et al, 1998) and a Boxer with mammary cancer containing an internal deletion within the p53 gene (patient 9, this study). Both of these breed types are found to display a high cancer frequency in the canine population and the characterization of these patients supports a link between heritable p53 gene mutations and breed predisposition to cancer. A more extensive analysis of the p53 gene status in breeds at greater risk for cancer will help to determine the relationship between germ line p53 mutations and cancer predisposition in the dog. Such information will be invaluable for breeding out cancer predisposition in identified lineages.

In addition to germline deletion in one p53 allele, patient 9 also displayed a germline base pair substitution at codon 69 in the second p53 allele (codon 82 in human p53). This substitution results in a codon change of proline to leucine and was also observed in the p53 sequence isolated from the tumour tissue of patient 6. Whether this sequence modification represents a polymorphism within the proline-rich region of canine p53 or simply a point mutation share by patients 6 and 9 remains unclear. However, preliminary analysis in our laboratory of the p53 status in an independent group of canine cancer patients lends support for the assignment of Pro69Leu as a p53 sequence polymorphism (data not shown).

A number of sequence polymorphisms occur in the human p53 gene including base pair substitutions at codons 21, 36, 47, 72, 148 and 213 (Béroud and Soussi, 1998). The most common p53 sequence polymorphism in humans is Arg/Pro at codon 72 (Ara et al, 1990). Recent reports indicate that individuals homozygous for Arg 72 may be predisposed to non-small-cell lung carcinoma (Tagawa et al, 1998) and/or human papillomavirus-associated cervical cancer (Storey et al, 1998). A similar situation could exist within canine cancer where the polymorphic status of the p53 gene (for example Pro69Arg of patient 9) represents a cancer risk factor. However, independent human studies have argued against a significant association between the p53 polymorphic status at position 72 and predisposition to cervical cancer (Helland et al, 1998; Minaguchi et al, 1998; Rosenthal et al, 1998) and the role, if any, that p53 polymorphisms play in tumorigenesis is uncertain.

© 1999 Cancer Research Campaign 

In summary, three of the ten canine cancer patients were found to contain mutations in the p53 gene (Figure 6). Tumour-associated p53 mutations were identified in patients 5 and 6, while germ line modifications were observed in both p53 alleles derived from patient 9. These results contribute to our understanding of the role of p53 inactivation in canine neoplasia and highlight the strong parallels between the molecular basis of cancer in dogs and humans. Moreover, our observations help to identify p53 inactivation as a possible factor in breed predisposition to cancer.

### ACKNOWLEDGEMENTS

We thank Meg Stark for photographic work and Mrs D Stanton for expert technical assistance. This work was supported by grant Y229PG from Yorkshire Cancer Research to JM.

### REFERENCES

Ara S, Lee PS, Hansen MF and Saya H (1990) Codon 72 polymorphism of the TP53 gene. *Nucleic Acids Res* **18**: 4961

Berns EM, Klijn JG, Smid M, van Staveren IL, Look MP, van Putten WL and Foekens JA (1996) TP53 and MYC gene alterations independently predict poor prognosis in breast cancer patients. *Genes Chromosomes Cancer* **16**: 170–179

Béroud C and Soussi T (1998) p53 gene mutation: software and database. *Nucleic Acids Res* **26**: 200–204

Birch JM, Blair V, Kelsey AM, Evans DG, Harris M, Tricker KJ and Varley JM (1998) Cancer phenotype correlates with constitutional TP53 genotype in families with the Li–Fraumeni syndrome. *Oncogene* **17**: 1061–1068

Casey G, Lopez ME, Ramos JC, Plummer SJ, Arboleda MJ, Shaughnessy M, Karlan B and Slamon DJ (1996) DNA sequence analysis of exons 2 through 11 and immunohistochemical staining are required to detect all known p53 alterations in human malignancies. *Oncogene* **13**: 1971–1981

Chang H, Benchimol S, Minden MD and Messner HA (1994) Alterations of p53 and c-myc in the clonal evolution of malignant lymphoma. *Blood* **83**: 452–459

Chu LL, Rutteman GR, Kong JMC, Ghahremani M, Schmeing M, Misdorp W, van Garderen E and Pelletier J (1998) Genomic organization of the canine p53 gene and its mutational status in canine mammary neoplasia. *Breast Cancer Res Treat* **50**: 11–25

Cook A and Milner J (1990) Evidence for allosteric variants of wild-type p53, a tumour suppressor protein. *Br J Cancer* **61**: 548–552

Devilee P, van Leeuwen IS, Voesten A, Rutteman GR, Vos JH and Cornelisse CJ (1994) The canine p53 gene is subject to somatic mutations in thyroid carcinoma. *Anticancer Res* **14**: 2039–2046

Faille A, De Cremoux P, Extra JM, Linares G, Espie M, Bourstyn E, De Rocquancourt A, Giacchetti S, Marty M and Calvo F (1994) p53 mutations and overexpression in locally advanced breast cancers. *Br J Cancer* **69**: 1145–1150

Gamble J and Milner J (1988) Evidence that immunological variants of p53 represent alternative protein conformations. *Virology* **162**: 452–458

Gannon JV, Greaves R, Iggo R and Lane DP (1990) Activating mutations in p53 produce a common conformational effect. A monoclonal antibody specific for the mutant form. *EMBO J* **9**: 1595–1602

Glebov OK, McKenzie KE, White CA and Sukumar S (1994) Frequent p53 gene mutations and novel alleles in familial breast cancer. *Cancer Res* **54**: 3703–3709

Hainaut P, Soussi T, Shomer B, Hollstein M, Greenblatt M, Hovig E, Harris CC and Montesano R (1997) Database of p53 gene somatic mutations in human tumors and cell lines: updated compilation and future prospects. *Nucleic Acids Res* **25**: 151–157

Hartmann A, Blaszyk H, Kovach JS and Sommer SS (1997) The molecular epidemiology of p53 gene mutations in human breast cancer. *Trends Genet* **13**: 27–33

Helland Å, Langerød A, Johnsen H, Olsen AO, Skovlund E and Børresen-Dale A-L (1998) p53 polymorphism and risk of cervical cancer. *Nature* **396**: 530–531

Hirano Y, Yamato K and Tsuchida N (1995) A temperature sensitive mutant of the human p53, Val138, arrests rat cell growth without induced expression of *cip1*/*waf1*/*sdi1* after temperature shift-down. *Oncogene* **10**: 1879–1885

Huang Y, Yin J and Meltzer SJ (1994) A unique p53 intragenic deletion flanked by short direct repeats results in loss of mRNA expression in a human esophageal carcinoma. *Carcinogenesis* **15**: 1653–1655

Johnson AS, Couto CG and Weghorst CM (1998) Mutation of the p53 tumor suppressor gene in spontaneously occurring osteosarcomas of the dog. *Carcinogenesis* **19**: 213–217

Kussie PH, Gorina S, Marechal V, Elenbaas B, Moreau J, Levine AJ and Pavletich N (1996) Structure of the MDM2 oncoprotein bound to the p53 tumor suppressor transactivation domain. *Science* **274**: 948–953

Lin J, Wu X, Chen J, Chang A and Levine AJ (1995) Functions of the p53 protein in growth-regulation and tumor suppression. *Cold Spring Harbor Symp Quant Biol* **59**: 215–223

MacVean DW, Monlux AW, Anderson PS Jr, Silberg SL and Roszel JF (1978) Frequency of canine and feline tumors in a defined population. *Vet Pathol* **15**: 700–715

Malkin D (1994) Germline p53 mutations and heritable cancer. *Annu Rev Genet* **28**: 443–465

Martinez J, Georgoff I, Martinez J and Levine A (1991) Cellular localization and cell cycle regulation by a temperature-sensitive p53 protein. *Genes Dev* **5**: 151–159

Mayr B, Schellander K, Schleger W and Reifinger M (1994) Sequence of an exon of the canine p53 gene-mutation in a papilloma. *Br Vet J* **150**: 81–84

Mayr B, Schaffner W, Botto I, Reifinger M and Loupal G (1997) Canine tumour suppressor gene p53-mutation in a case of adenoma of circumanal glands. *Vet Res Comm* **21**: 369–373

Mayr B, Dressler A, Reifinger M and Feil C (1998) Cytogenetic alterations in eight mammary tumors and tumor-suppressor gene p53 mutation in one mammary tumor from dogs. *Am J Vet Res* **59**: 69–78

Minaguchi T, Kanamori Y, Matsushima M, Yoshikawa H, Taketani Y and Nakamura Y (1998) No evidence of correlation between polymorphism at codon 72 of p53 and risk of cervical cancer in Japanese patients with human papillomavirus 16/18 infection. *Cancer Res* **58**: 4585–4586

Moll UM, Riou G and Levine AJ (1992) Two distinct mechanisms alter p53 in breast cancer: mutation and nuclear exclusion. *Proc Natl Acad Sci USA* **89**: 7262–7266

Priester WA and McKay FW (1980) The occurrence of tumors in domestic animals. *Natl Cancer Inst Monograph* **54**: 152–184

Rolley N, Butcher S and Milner J (1995) Specific DNA binding by different classes of human p53 mutants. *Oncogene* **11**: 763–770

Rosenthal AN, Ryan A, Al-Jehani RM, Storey A, Harwood CA and Jacobs IJ (1998) p53 codon 72 polymorphism and risk of cervical cancer in UK. *Lancet* **352**: 871–872

Seth A, Palli D, Mariano JM, Metcalf R, Venanzoni M, Bianchi S, Kottaridis SD and Papas TS (1994) P53 gene mutations in women with breast cancer and a previous history of benign breast disease. *Eur J Cancer* **30A**: 808–812

Storey A, Thomas M, Kalita A, Harwood C, Gardiol D, Mantovani F, Breuer J, Leigh IM, Matlashewski G and Banks L (1998) Role of a p53 polymorphism in the development of human papillomavirus-associated cancer. *Nature* **393**: 229–234

Strauss BS (1997) Silent and multiple mutations in p53 and the question of the hypermutability of tumors. *Carcinogenesis* **18**: 1445–1452

Sun X-F, Johannsson O, Håkansson S, Sellberg G, Nordenskjöld B, Olsson H and Borg A (1996) A novel p53 germline alteration identified in a late onset breast cancer kindred. *Oncogene* **13**: 407–411

Tagawa M, Murata M and Kimura H (1998) Prognostic value of mutations and germ line polymorphism of the p53 gene in non-small cell lung carcinoma: association with clinicopathological features. *Cancer Lett* **128**: 93–99

Tornaletti S and Pfeifer GP (1995) Complete and tissue-independent methylation of CpG sites in the p53 gene: implications for mutations in human cancers. *Oncogene* **10**: 1493–1499

van Leeuwen IS, Hellmen E, Cornelisse CJ, van den Burgh B and Rutteman GR (1996) P53 mutations in mammary tumor cell lines and corresponding tumor tissues in the dog. *Anticancer Res* **16**: 3737–3744

van Leeuwen IS, Cornelisse CJ, Misdorp W, Goedegebuure SA, Kirpensteijn J and Rutteman GR (1997) P53 gene mutations in osteosarcomas in the dog. *Cancer Lett* **111**: 173–178

Veldhoen N and Milner J (1998) Isolation of canine p53 cDNA and detailed characterization of the full length canine p53 protein. *Oncogene* **16**: 1077–1084

Veldhoen N, Stewart J, Brown R and Milner J (1998) Mutations of the p53 gene in canine lymphoma and evidence for germ line p53 mutations in the dog. *Oncogene* **16**: 249–255

Venkatachalam S, Shi Y-P, Jones SN, Vogel H, Bradley A, Pinkel D and Donehower LA (1998) Retention of wild-type p53 in tumors from p53 heterozygous mice:

© 1999 Cancer Research Campaign reduction of p53 dosage can promote cancer formation. *EMBO J* **17**: 4657–4667

Wada H, Asada M, Nakazawa S, Itoh H, Kobayashi Y, Inoue T, Fukumuro K, Chan LC, Sugita K, Hanada R, Akuta N, Kobayashi N and Mizutani S (1994) Clonal expansion of p53 mutant cells in leukemia progression in vitro. *Leukemia* **8**: 53–59

Yamato K, Yamamoto M, Hirano Y and Tsuchida N (1995) A human temperature-sensitive p53 mutant p53val-138: modulation of the cell cycle, viability and expression of p53-responsive genes. *Oncogene* **11**: 1–6

*© 1999 Cancer Research Campaign*
